# Age-Related Left Ventricular Changes and Their Association with Leukocyte Telomere Length in Healthy People

**DOI:** 10.1371/journal.pone.0135883

**Published:** 2015-08-14

**Authors:** Dariga U. Akasheva, Ekaterina V. Plokhova, Olga N. Tkacheva, Irina D. Strazhesko, Ekaterina N. Dudinskaya, Anna S. Kruglikova, Valentina S. Pykhtina, Natalia V. Brailova, Inna A. Pokshubina, Natalia V. Sharashkina, Mikhail V. Agaltsov, Dmitry Skvortsov, Sergey A. Boytsov

**Affiliations:** 1 Department of study the ageing and prevent age-associated diseases, National Research Center for Preventive Medicine, Moscow, Russian Federation; 2 Department of Chemistry, Lomonosov Moscow State University, Moscow, Russian Federation; INRCA, ITALY

## Abstract

**Introduction:**

With advancing age the left ventricle (LV) undergoes structural and functional changes, thereby creating the substrate for the development of diseases. One possible mechanism of the ageing heart is a cellular senescence. Leukocyte telomere length (LTL) is a marker of replicative ageing. The purpose of this study was to evaluate the structure and function of the LV in people of different ages free of cardiovascular diseases (CVD) and regular drug medication and to assess their relationship with LTL. We hypothesized that age-related changes in LV myocardium are associated with telomere length.

**Methods:**

The study population consisted of 150 healthy, non-obese volunteers aged 28 to 78 years without history of CVD, significant deviations by 12-lead electrocardiogram and negative exercise test (treadmill stress test). All the participants underwent standardized transthoracic echocardiography using an available system (iE33; Philips). The LTL was measured by real-time quantitative polymerase chain reaction. We determined the relative ratio of telomere repeat copy number (T) to single-copy gene copy number (S).

**Results:**

In the older people there was a higher wall thickness than in the younger (1.03±0.09 vs. 0.88±0.10, p<0.01), whereas LV mass index was comparable between them (85.8±15.40 vs. 83.1±11.8, p = 0.20). There was a decrease in LV dimensions with advancing age (p<0.001). Older subjects had impairment in LV relaxation. LTL was associated with decreased E/A, Em/Am ratio (β = -0.323, p = 0.0001) after adjusting for age, sex and risk factors. There is no relation between the LTL and the structure of LV.

**Conclusions:**

Our data suggest that the ageing process leads to changes in LV structure and diastolic function and is linked with a phenotype of concentric LV remodeling. Telomere attrition is associated with age-related LV diastolic dysfunction. Telomere length appears to be a biomarker of myocardial ageing.

## Introduction

There are a number of structural and functional changes in the heart with ageing in people without diseases and each of these can have significant implications for cardiovascular disease (CVD). Conflicting data have been accumulated regarding the structural changes of LV. However, age-related impairments of the LV functions are well described. Despite maintenance of systolic function at rest, there are changes in the diastolic phase of the cardiac cycle that occur with ageing [1].

In addition, the interest of scientists aims to identify genetic markers that allow evaluation of age-related changes of the cardiovascular system. Although the relation of advancing age with diastolic dysfunction is widely recognized, their association with cellular senescence is less clear. Telomere length has been regarded as a marker of biologic ageing. Telomeres are repetitive DNA sequences (*TTAGGG*) located at the ends of chromosomes which protect DNA from damage. Telomeres progressively shorten in somatic cells with increasing number of cell divisions. When the telomeres become critically short, the cell is no longer able to replicate and enters cellular senescence [2].

Cardiac tissue from animal models shows substantial loss of telomere length with age and telomerase knockout in mice lead to heart failure, suggesting a possible role for telomere shortening in the development of heart ageing [3,4]. In the present study, we examined telomere length in leukocytes. Studies have shown that the intra-individual correlation between telomere lengths in different tissues is high [5]. The association of leukocyte telomere length (LTL) with the other tissues was also consistently high [6]. The average LTL is inversely correlated with age and is associated with age-related disorders, including CVD [7]. Therefore, telomere length in accessible tissues such as blood, can serve as a surrogate parameter for determining the relative telomere length in other tissues. However, the importance of the cellular ageing processes for determining myocardial function is presently little studied.

In individuals free of CVD, we sought to comprehensively assess age-related changes in the structure and function of the LV using echocardiography, and also to determine if these changes were associated with a marker of cellular senescence.

## Materials and Methods

### Study participants

By advertisement we recruited subjects who visited the National Research Center for Preventive Medicine in Moscow, Russia, from May 2012 to May 2013. All participants completed a health screening, which included their medical history, a physical examination. Subjects who had symptoms or a history of CVD (including hypertension II and III degrees, stroke, coronary heart disease, peripheral arterial disease, arrhythmia, congestive heart failure, valvular heart disease) or took any regular medications for CVD; diabetes, thyroid disease, hepatic or kidney failure, cancer, pregnancy and lactation were excluded. As a result, 240 participants aged 23–91 years were selected.

Consenting subjects had a physical assessment including blood pressure (BP) determination, mmHg (using sphygmomanometer HEM-7200 M3, Omron Healthcare), heart rate, anthropometric measurements (body mass index (BMI); kg/m^2^), a blood sampling for laboratory analyses, electrocardiogram recording and a treadmill stress test. A treadmill stress test was performed on a protocol BRUCE using available system (Intertrack, SCHILLER). Patients with important abnormalities (such as BMI≥30 kg/m^2^, significant deviations in laboratory analyses, rhythm and conduction disorders in the electrocardiogram or a positive exercise test result) were excluded. Subsequently, the study population consisted of 150 participants. Each subject underwent full echocardiographic examination and measurement of LTL. The study protocol was approved by the institutional Ethics Committee, and all participants gave written informed consent.

### Echocardiography

Transthoracic 2D echocardiography was performed with the available system (iE33; Philips). All echocardiographic measurements were averaged from 3 cardiac cycles. The following data were acquired: LV end-diastolic septal (SWT) and posterior wall thickness (PWT), LV diameter at the end of diastole (LVDD) and LV diameter at the end of systole (LVDS); LV volumes; LV mass index (LVMI) and LV diastolic function parameters. LV linear dimensions were measured from a parasternal long-axis view. LV mass was calculated by the area-length method and indexed to body surface area to obtain LVMI. Quantitative 2D methods (biplane Simpson’s) were performed to obtain an ejection fraction (EF).

Doppler-echocardiographic mitral valve inflow velocities were recorded from an apical 4-chamber view with a cursor at the tips of the mitral valve leaflets. Peak velocity of early filling (E) and peak velocity of atrial filling (A) were measured and the E/A ratio was calculated. To measure isovolumic relaxation time (IVRT), the sample volume was placed between the anterior leaflet of the mitral valve and LV outflow tract and the E wave deceleration time (DTE) was calculated as the time between the peak of early filling and the upper deceleration slope extrapolated to the baseline [8].

Pulsed Doppler of pulmonary venous flow was performed in the apical 4-chamber view with location of the sample volume in the right upper pulmonary vein. Peak systolic (S) velocity, peak anterograde diastolic (D) velocity, the S/D ratio, peak retrograde (PV Ar) velocity in late diastole and its duration (tApv) were evaluated [8].

LV myocardial velocities were evaluated by tissue Doppler imaging (TDI). Pulsed TDI sample volume was placed at the level of the lateral mitral valve annulus in the apical 4-chamber view. Early diastolic (Em) and late diastolic (Am) velocities were measured, and Em/Am, E/Em ratios were calculated [9].

### Measurement of LTL

LTL was determined according to the method described by Cawthon [10]. Genomic deoxyribonucleic acid (DNA) was extracted directly from blood samples by standard procedures (OD260nm/280nm 1.8–1.9). The assay involved comparing the abundance of telomere DNA to the single copy genomic DNA number for each sample and by further comparison of normalized value between DNAs of different sources. The ratio of the telomere (T) and single-copy 36B4 gene (S) matrices reflect the length of telomeres (**S1 File**).

### Statistical analysis

The statistical analysis was performed by SAS 9.1 (SAS Institute, Cary, NC, USA). The intergroup comparisons were performed using Student’s t-test or ANOVA when appropriate. Data are presented as means ± SD for continuous variables and as proportions for categorical variables. The association LTL with LV structure and function parameters were evaluated using correlation analysis (Spearman’s and Pearson coefficients), linear and multivariate regression analysis. We also used logistic regression models to estimate odds ratios and χ^2^. The level of statistical significance was set at p<0.05.

## Results

One hundred and fifty individual (62 males and 88 females) was accepted. The subjects ranged between 28 and 78 years of age, with a mean age of 51.3±12.3 years. Of the study group, 31 subjects had mild arterial hypertension, 27 subjects was overweight (mean BMI 26.7±6.0 kg/m^2^) and 28 were smokers. The duration of hypertension was less than 0.6±0.3 years. None of the participant regularly received any other medication, including antihypertensive drugs. Table 1 displays the clinical and echocardiographic characteristics of the study sample overall.

**Table 1:** Comparison of the main clinical, echocardiographic characteristics and leukocyte telomere length between younger and older individuals.

*Characteristics*	*Group 1 (n = 78) 28–59 years*	*Group 2 (n = 72) 60–78 years*	*p*
*Clinical characteristics*			
Age, years	39±7.9	65±5.3	< 0.001
Male, n (%)	33 (42)	29 (40)	0.15
Body mass index, kg/m^2^	26±4.10	27±2.59	0.10
Systolic blood pressure, mm Hg	128±12.47	133±12.9	0.50
Diastolic blood pressure, mm Hg	76±9.94	77±7.80	0.60
Smokers, n (%)	17 (13)	20 (14)	0.8
*Echocardiographic characteristics*			
LVDD, cm	4.94±0.37	4.4±1.07	0.001
LVSD, cm	2.68±0.15	2.47±0.94	0.10
LVDV, ml	80±19.02	64.6±11.79	< 0.001
LVSV, ml	29±6.89	23±6.34	< 0.001
LVEF, %	61±2.40	63±3.10	0.13
SWT, cm	0.88±0.10	1.03±0.09	< 0.01
PWT, cm	1.01±0.22	1.11±0.33	< 0.001
LVMI, g/m^2^	83.1±11.8	85.8±15.40	0.20
E/A ratio	1.27±0.28	0.96±0.31	< 0.001
IVRT, ms	70±10.90	81±13.22	< 0.001
DTE, ms	175±24.90	198±33.80	< 0.001
Em lateral, cm/s	13.1±3.14	9.3±0.77	< 0.001
Am lateral, cm/s	9.2±2.05	10.7±2.36	0.002
E/Em ratio lateral	5.4±1.34	7.1±1.69	< 0.001
Em/Am ratio lateral	1.5±0.58	0.9±0.30	< 0.001
PV S/D ratio	1.0±0.04	1.3±0.21	< 0.001
PV Ar, cm/s	28.9±5.45	30.6±0.51	< 0.01
PV Ar duration, ms	102±23.80	119±23.05	< 0.001
*Marker of cellular senescence*			
Relative LTL	9.89±0.30	9.70±0.56	0.02

Measurements are shown as means ± SD. *LVDD*, LV diameter at the end of diastole; *LVDS*, LV diameter at the end of systole; *LVDV*, LV end-diastolic volume; *LVSV*, end-systolic volume; *LVMI*, LV mass index; *EF*, ejection fraction; *SWT*, septal wall thickness; *PWT*, posterior wall thickness; *E*, peak early phase filling velocity; *A*, peak atrial phase filling velocity; *DTE*, E wave deceleration time; *IVR*T, isovolumic relaxation time; *Em*, peak early diastolic mitral annular velocity; *Am*, peak diastolic mitral annular velocity; *S*, peak systolic velocity of pulmonary venous flow; *D*, peak anterograde diastolic velocity of pulmonary venous flow; *PV Ar*, peak retrograde velocity in late diastole of pulmonary venous flow.

Study subjects were categorized according to age into 2 groups, of 28 to 59 years (group 1, n = 78) and of 60 to 78 years (group 2, n = 72). The mean age was 39±7.9 year and 65±5.3 year, respectively (p<0.001). There were no significant differences in the sex, BMI and the levels of BP between the groups. SWT and PWT were larger in older individuals (p<0.01), but there was no difference between groups in LVMI (p = 0.2). Older subjects had lower values of LVDD, LV end-diastolic volume (LVDV) and end-systolic volume (LVSV).

Analysis of the Doppler indices revealed significant differences between groups: E/A velocity ratio (0.96±0.31 vs 1.27±0.28) were lower in older subjects with a significant increase in IVRT (81±13.22 ms vs 70±10.90 ms) and DTE (198±33.80 ms vs 175±24.90 ms). The assessment of pulmonary venous flow showed significantly greater values of the S/D ratio (1.3±0.21 vs 1.0±0.04), PV Ar (30.6±0.51 cm/s vs 28.9±5.45 cm/s) and its duration (119±23.05 ms vs 102±23.80 ms) in a group of older people. Table 1 also shows the mitral annular velocities measured with TDI. We found lower early diastolic Em (9.3±0.77 cm/s vs 13.1±3.14 cm/s) velocities and Em/Am ratio (p<0.001) in the group of older subjects, while late diastolic Am velocity and the E/Em ratio was greater (p<0.001).

Median of leukocyte telomere length was 9.75. Telomeres were termed “short” if the LTL was <9.75, and “long” if the LTL was ≥9.75. Age was significantly related to telomere length (β = -0.012, p = 0.001). There is no relation between the LTL and the structure of LV. We evaluated associations between LTL and LVMI (r = -0.21, p<0.01), SWT (r = -0.25, p = 0.01), LVDV (r = 0.10, p>0.05) and LVSV (r = 0.12, p>0.05). Correlation LTL with LVMI and SWT was weak. According to the logistic regression model, we have not received significant relation LTL with these parameters: SWT (χ^2^ = 1.77, p = 0.18, OR = 1.44; 95% CI 0.84–2.47), LVMI (χ^2^ = 0.37, p = 0.55; OR = 1.56; 95% CI 0.37–6.59).

**Fig 1:**
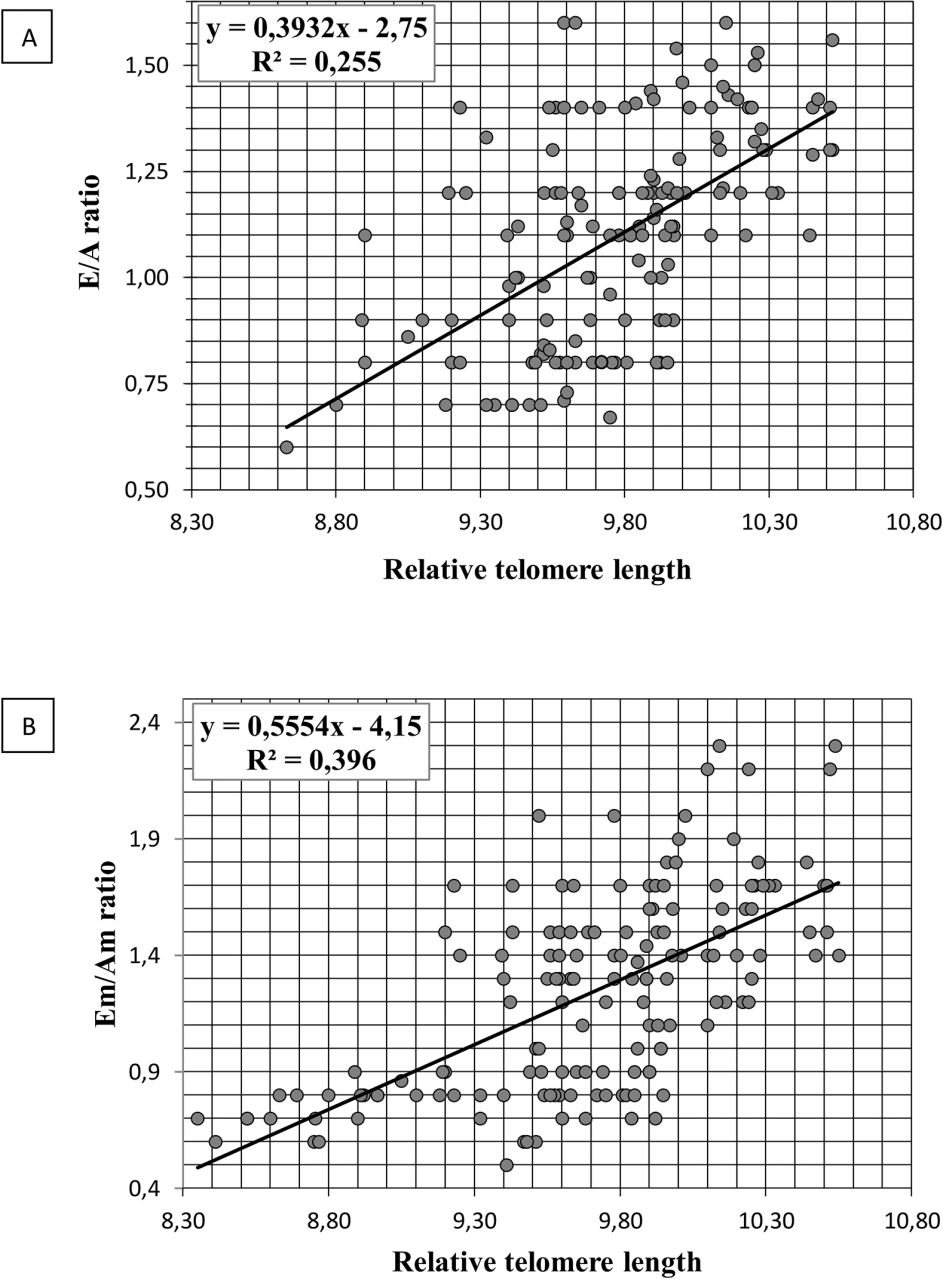
Scatter plots showing the linear relationships between age and leukocyte telomere length, indices of LV diastolic function and leukocyte telomere length. (A) Linear regressions between E/A ratio and leukocyte telomere length. (B) Linear regressions between Em/Am ratio and leukocyte telomere length.

There was a positive correlation between telomere length and E/A ratio (r = 0.3; p<0.01), Em/Am ratio (r = 0.36; p<0.05), E′ (r = 0.34, p<0.001), IVRT (r = -0.33, p<0.001). Linear regression analysis showed comparable changes of Doppler measurements (E/A and Em/Am ratio) with increase LTL (Fig 1A and 1B).

In multivariate regression analysis LTL was associated with decreased E/A, Em/Am ratio (β = -0.323, p = 0.0001) after adjusting for age, sex and risk factors. Shorter telomere was associated with an increased risk of reducing the E/A (χ^2^ = 52.07, p = 0.001; OR = 24.95; 95% CI 10.41–59.77) and Em/Am ratio (χ^2^ = 45.37, p = 0.001; OR = 11.99; 95% CI 5.82–24.70). The lowest value of telomere length within the first quintile increase the risk of LV diastolic dysfunction (reduction of E/A, Em/Am, Em and an increase in IVRT) in 55 times (OR = 55.1; p = 0.0001). In older people with length of telomeres <9.75 diastolic dysfunction was diagnosed in 92% of cases. While in older adults with telomere length ≥ 9.75 was at 42% only. According to our expectations «short» LTL is a predictor of age-related changes in LV diastolic dysfunction and is associated with an early decrease in E/A and Em/Am ratio. Older individuals with “long” telomeres had higher values of these parameters than older subjects with “short” telomeres.

## Discussion

In a sample of uneven aged individuals free of CVD of 28 to 78 years we initially examined the structural and functional age-related changes to the LV. Comprehensive assessment of cardiac remodeling in the people without CVD has been limited and sometimes contradictory. Several studies have shown no changes in LV wall thickness [11, 12] and dimensions with ageing [12]. Some authors consider that diastolic chamber dimension and LV mass increases with advancing age [13,14], while others show its decline [15, 16]. Our findings indicated that LV wall thickness was greater in the older people, whereas LV mass did not change in healthy adults. Furthermore, the LV internal dimension in diastole and its volumes were less in older than in younger adults. Thus, according to our data and other studies [17], ageing is associated with a phenotype of concentric LV remodeling. This type of age-based ventricular remodeling is likely related to the coupling of ventricular and vascular stiffening processes that can occur over a lifetime [1, 15]. Concentric LV remodeling conferred significant risk of cardiovascular events [15], therefore, the identification of such structural changes in older people is very important. Age-related changes in LV structure are often accompanied by functional disorders. The overall resting systolic function of LV as a rule does not change with healthy ageing [12]. Our results also showed the stability of cardiac EF at rest. Studies in healthy human beings have documented that ageing is associated with a progressive decline in diastolic function [18]. Diastolic dysfunction with preserved EF accounts for 50% of heart failure cases in population-based studies [19]. In this study, we evaluated all the known parameters of diastolic function. It was demonstrated impairment in LV relaxation in older people. Tissue Doppler indices appeared more sensitive to ageing. The deterioration in relaxation of the longitudinal myocardial fibers occurs before the decline in the global diastolic function. The changes in diastolic function with age have been well described. Therefore, our main objective was to assess the association between age-related changes in LV diastolic function and cellular senescence.

We tested the hypothesis that LTL is a marker of biological ageing of LV. The association between LTL and age-related structural changes of the myocardium were not obtained in our study. Additional logistic regression analysis was inconclusive. Previous studies have yielded controversial results. So, in the Framingham study a positive relationship called by some paradoxical has been found: subjects with LV myocardial hypertrophy have longer telomeres [20]. The lack of reliable association LTL with LVMI in our study can be explained by included of people with healthy hearts without hypertrophy. In the Asklepios study population free from overt cardiovascular disease also has not been found significant relations between LTL and LVMI [21].

We analyzed the association between the LTL and LV diastolic function in different age groups. We used the most sensitive to the age parameters of LV diastolic function—Em/Am ratio and E/A. Our results showed that LTL positively correlated with LV diastolic function. We observed a significant and independent of age relationship between LTL and LV diastolic indices. In the same age group, the presence of shorter telomeres contributed to a greater LV diastolic dysfunction. This suggests that ageing may affect myocardial function and the responsible mechanism is the replicative senescence, which related to shortening telomere length with age.

A possible explanation for age-associated LV remodeling and LV diastolic dysfunction is changing at the cellular level. From the age of 17 to the age of 90 years, 30–35% of myocardial cells are lost. The cell loss leads to hypertrophy of the adjacent myocytes, resulting in an increase of ventricular wall thickness. However, this cellular hypertrophic response is unable to increase cardiac mass [15]. In parallel, there is an increase in interstitial collagen content, fibrosis and depositions of ‘senile’ cardiac amyloid, which lead to increased myocardial stiffness and decrease the compliance [22].

These changes are caused by cellular ageing. One theory of ageing cells is based on telomere shortening. Many human tissues showed telomere attrition with age [5]. The primary determinant of telomere length is inherited, but it is also influenced by environmental factors, making it a valuable biomarker for human ageing [23]. In our study, significant association LTL with participants’ age was obtained. Shorter telomeres were associated with older age.

The association between telomere length and an ageing myocardium is little studied. Previously it was considered that all cardiomyocytes were terminally differentiated cell type unable to undergo mitosis [22]. Therefore, the telomeres length should not change with age in these cells and one possible mechanism responsible for cardiac age-related change is likely to be apoptosis. Recent experiments discovered proliferation and cell division in a fraction of cardiomyocytes in the adult heart [24]. The source of new cardiomyocytes could be cardiac stem cells. The cardiomyocytes and progenitor cardiac cells showed a reduction in telomere length with age. Accordingly, the story of the ageing myocardium has changed.

M. Terai et al. demonstrated a yearly telomere reduction rate of 20 base pairs in the myocardium. There was a significant correlation between myocardial telomere and ageing. Moreover, a small but significant correlation between telomere reduction and heart weight was demonstrated. Hearts of autopsied patients who had died of heart disease were significantly heavier than those of patients who had died of cancer or other diseases, and heart disease was significantly more correlated with myocardial telomere shortening than cancer or other diseases [25].

Telomere shortening in cardiomyocytes leads to their ageing. The accumulation of senescent cells with age contributes to the development phenotype of an ageing heart. Telomere shortening in mice was associated with attenuation in cardiac myocyte proliferation, increased apoptosis and cardiac myocyte hypertrophy, suggesting a possible role for telomere shortening in the development of a phenotype of the old heart. In mouse with shorter telomeres were observed a decrease of left ventricular mass, impaired relaxation and ventricular contractility, as well as the development of heart failure [3]. A study of 67 older people men revealed a positive association LTL with LVEF within its normal range, however, the association with parameters of LV diastolic function is not proven. These data show influences of the ageing process on myocardial function [26].

We acknowledge several limitations to this study. The sample size was restricted, and larger studies are needed to confirm these results. This is cross-sectional study and a number of factors that may have affected telomere attrition were not considered in these analyses, including lifestyle and other health behaviors.

In conclusion, the present study demonstrates that normal cardiac ageing related to a moderate LV wall thickening without change in LV mass, decrease in LV dimensions and progressive decline in diastolic function. We have also shown that LTL, a biomarker of ageing, is independent of age associated with LV diastolic function parameters. This suggests that telomere length appears to be a biomarker of cardiac ageing. Prospective longitudinal studies are needed for understanding of the relationship between telomere length and cardiovascular ageing.

## Supporting Information

S1 File Measurement of LTL. (PDF)
