# Supplementary material for: Age-Related Left Ventricular Changes and Their Association with Leukocyte Telomere Length in Healthy People
Source: PLoS One. 2015 Aug 14;10(8):e0135883. doi: 10.1371/journal.pone.0135883 (PMC4537122; doi:10.1371/journal.pone.0135883)
Supplement: S1 File — (PDF) [file pone.0135883.s001.pdf]

Supporting Information S1

Measurement of LTL

The T/S ratio is approximately [2Ct (telomeres)/2Ct(36B4)]–1=2–ΔCt [T1]. Simultaneously stock mix 1,25 x (1x mixture: PCR buffer 1x (Fermentas 10X PCR Hotstart buf + KCl), MgCl2 2 mM, dNTP 0.2 mM, 0.5 μM of each primer, 0.05 units / μl of Taq polymerase Maxima (Fermentas), Sybr Green I 0.2x) have been prepared. The primer sequences were: Tel1, GGTTTTTGAGGGTGAGGGTGAGGGTGAGGGTGAGGGT,
Tel2, TCCCGACTATCCCTATCCCTATCCCTATCCCTATCCCTA.,
36B4u, CAAGTGGGAAGGTGTAATCC, 36B4d, CCCATTCTATCATCAACGGGTACAA. Sixteen microliters of master mix were added to each sample well and 4 μl of the analyzed genomic DNA with a concentration of 10 ng/μl was added. Samples were mixed, centrifuged, and amplified in a thermocycler CFX96. For telomere polymerase chain reaction (PCR), we then heated at 95C for 5 minutes, and did 35 cycles of 95C for 20 sec, 54C for 2 minutes, followed by melting. For control PCR we heated at 95 C for 5 minutes. Then we did 35 cycles of 95C for 20 sec, 58C for 1 minute, followed by melting. The amplification of the corresponding telomeric and control mixtures occupy one cell unit. For each sample, we did three repetitive telomeric reactions and three control reactions. We calculated the difference between cycle thresholds of amplification of the telomere and single copy of the gene (ΔCt), and based on these results appreciated relative telomere lengths. The genomic DNA of the cell line HEK293T and control leukocyte sample was used as a reference point. To take into account differences in PCR mixtures from time to time the we set the leukocyte reference ΔCt(leu) value at 8. Relative exponential length L was set L=ΔCt-(ΔCt(leu)-8). As we do not get the absolute value of the lengths of telomeres, so as a measure of the spread of values, it was decided to use the standard deviation. Each analyzes we perform from two to four times to calculate standard deviation. In our experiment, the standard deviation in almost all cases was in the range of 0.1-0.4 derived from the relative lengths of 8.30 to 11.39 (logarithmic scale).
